# Real-Time EMG Based Pattern Recognition Control for Hand Prostheses: A Review on Existing Methods, Challenges and Future Implementation

**DOI:** 10.3390/s19204596

**Published:** 2019-10-22

**Authors:** Nawadita Parajuli, Neethu Sreenivasan, Paolo Bifulco, Mario Cesarelli, Sergio Savino, Vincenzo Niola, Daniele Esposito, Tara J. Hamilton, Ganesh R. Naik, Upul Gunawardana, Gaetano D. Gargiulo

**Affiliations:** 1 The MARCS Institute, Western Sydney University, Werrington 2747, NSW, Australia; Ganesh.Naik@westernsydney.edu.au; 2 School of Computing, Engineering and Mathematics, Western Sydney University, Penrith 2751, NSW, Australia; N.sreenivasan@westernsydney.edu.au (N.S.); U.Gunawardana@westernsydney.edu.au (U.G.); G.Gargiulo@westernsydney.edu.au (G.D.G.); 3 Department of Information Technology and Electrical Engineering, “Federico II” The University of Naples, Via Claudio 10, 80140 Naples, Italy; pabifulc@unina.it (P.B.); cesarell@unina.it (M.C.); sergio.savino@unina.it (S.S.); vincenzo.niola@unina.it (V.N.); daniele.esposito@unina.it (D.E.); 4 School of Engineering, Macquarie University, Macquarie Park (NSW), Waterloo road, Sydney 2113, Australia; tara.hamilton@mq.edu.au

**Keywords:** Myo-prosthesis, EMG, Pattern recognition, myosignals

## Abstract

Upper limb amputation is a condition that significantly restricts the amputees from performing their daily activities. The myoelectric prosthesis, using signals from residual stump muscles, is aimed at restoring the function of such lost limbs seamlessly. Unfortunately, the acquisition and use of such myosignals are cumbersome and complicated. Furthermore, once acquired, it usually requires heavy computational power to turn it into a user control signal. Its transition to a practical prosthesis solution is still being challenged by various factors particularly those related to the fact that each amputee has different mobility, muscle contraction forces, limb positional variations and electrode placements. Thus, a solution that can adapt or otherwise tailor itself to each individual is required for maximum utility across amputees. Modified machine learning schemes for pattern recognition have the potential to significantly reduce the factors (movement of users and contraction of the muscle) affecting the traditional electromyography (EMG)-pattern recognition methods. Although recent developments of intelligent pattern recognition techniques could discriminate multiple degrees of freedom with high-level accuracy, their efficiency level was less accessible and revealed in real-world (amputee) applications. This review paper examined the suitability of upper limb prosthesis (ULP) inventions in the healthcare sector from their technical control perspective. More focus was given to the review of real-world applications and the use of pattern recognition control on amputees. We first reviewed the overall structure of pattern recognition schemes for myo-control prosthetic systems and then discussed their real-time use on amputee upper limbs. Finally, we concluded the paper with a discussion of the existing challenges and future research recommendations.

## 1. Introduction

The human upper limb is a significant part of the body, the partial or complete loss of which can have a significant effect on a person’s ability on the day to day activities. The human upper limb has three sections the hand, forearm and arm. For the movement of each section, coordinating the relation of the nervous system, musculoskeletal systems and its surroundings are necessary. To perform various daily activities, coordination of different joints (shoulder, elbow, wrist and finger joint) is essential, including a broad range of motions with several degrees of freedom. These coordinated movements are always redundant and can be beneficial to perform complex tasks. When it comes to an artificial hand, all such control features of the normal hand should extensively match, so that the user can perform their daily needs in a modified and effective way. The coordinated control of the biological hand is quite complex, making it highly difficult to replicate it exactly in any prosthetic hand. 

A typical prosthetic hand involves three main connected parts: an input signal acquisition unit, processing and control unit and an end effector. Nowadays, almost all high performing artificial hands (or prosthesis) use surface electromyography signals (sEMG or myosignals) for controlling their end effectors. Surface electromyography records the muscle movements electrically from the surface of muscle cells when they are electrically or neurologically activated [1]. The amplitude of sEMG signals ranges from 0 to 10 mV (peak to peak)/0 to 1.5 mV (RMS) with dominant energy in the 20–450 Hz band [2]. Moreover, the acquisition of the sEMG signal requires proper skin preparation, and EMG electrodes should be placed after confirming the target muscles (from which the EMG signal comparable to predefined limb movement can be produced). With the technological and miniaturization of sensors, dry electrodes that work as transducers for muscular inputs have replaced traditional gel EMG electrodes and have improved performance [3]. On a usability level, sometimes, muscle fatigue can happen due to the positioning of these dry electrodes on a single target muscle [4]. Recently, a modular scheme was developed as a solution, which uses the combination of several electrodes and channels for accurate quantification [5,6]. An extension to this is that these electrodes are replaced with transducers, such as force and resistive sensors, which use only a single channel acquisition method with fewer disturbances [7,8].

In general, myoelectric hands have evolved a lot to overcome the traditional disadvantages of acquiring myosignals to satisfy the needs of all levels of amputees. However, the basic control of the majority of those myo-activated limbs has followed the same operating principles (muscle contractions) for more than half of a century [9,10]. These devices use two types of technical control: pattern recognition (PR)-based control and non-pattern recognition-based control [11]. The conventional non-pattern recognition method is commonly used and limited to the proportional control (on/off control). EMG-PR techniques have been developed to increase the dexterity of myoelectric prosthetic devices, and to overcome the limitations of conventional proportional control. EMG-PR operates by extracting multiple features from EMG signals rather than entirely relying on EMG amplitude [12] (as EMG amplitude is slow, cumbersome and difficult for users to control their residual muscles movement). A well-developed artificial upper limb design comprises of trajectories of a limb and their associated movement patterns. To delineate this, a control algorithm requires parameters such as kinematics and models of joints [13], motion and activities range [11]. Through EMG-based pattern recognition, researchers are working on the hypothesis that EMG patterns contain much information on intended movements. Once the EMG patterns are identified for intended movements using pattern classification, the prosthesis controller will receive the command to implement the movement. Thus, EMG-PR approach may allow users to control their myoelectric prosthesis more effortlessly with a broad range of control.

The use of artificial hands instead of biological hands with the same degree of dexterity [14] and complexity is a challenging task. However, pattern recognition (PR) technology has played an important role in controlling myoelectric prosthetic devices for over 20 years [15,16,17]. Pattern recognition technology provides more natural control, which is easier to learn by user and machine. It also provides independent control of multiple DOFs using simultaneous, sequential or semi-sequential control, as well as bringing the prosthesis closer to natural arm functions [18]. By applying proper PR-based methods and signal processing techniques in combination with machine learning algorithms, an amputee’s limb movement can be accurately decoded and used to control a prosthetic device [11,19]. EMG-based PR methods involve various approaches such as pre-processing, segmentation of data, feature extraction, feature classification and post-processing [20]. 

All these approaches related to myoelectric pattern recognition in one way or another can be helpful, but these methods still need further real-time evaluations for their validity [6]. Much research has been done on the myoelectric prosthesis; nevertheless, some of the areas in the field need to be improved: (i) control of multiple degrees of freedoms (DOFs) naturally and intuitively, (ii) two-way communication with the brain (peripheral nervous system (PNS)) and iii) fast learning. Moreover, several advanced pattern recognition techniques have been proposed without any real-world user applications [21,22]. A large portion of pattern recognition techniques described in the literature is still being applied in clinical settings. Moreover, the performance of these algorithms is affected greatly by several factors, including the positioning of electrodes, the fatigue of the muscle, arm position, surface EMG cross-talk and muscle contraction. This paper is mainly focused on a review of the major pattern recognition control approaches for myo-activated prostheses and their real-time amputee applications, and then suggests some critical directions to follow to improve their performance level while maintaining quantifiable viability.

The research methodology is discussed in Section 2. Section 3 describes the pattern recognition techniques and systems used today, their merits and demerits if these are hands-on and what extents need to be innovative for these technologies to be accomplished. Section 4 describes the methods and analysis of the real-time application of myo-prosthesis. The results are discussed in Section 5. The issues and advances made concerning this research area are discussed in Section 6. Section 7 concludes and provides an overall analysis of the article.

## 2. Research Methodology

This paper is a review article that summarises the current state of real-time EMG-PR control of hand prosthesis. Many classification approaches have been proposed to obtain better performance of the real-time application of myoelectric prosthesis. Despite the fact that the classification accuracy is high (nearly >95%) on offline measurements, the implementation of classification techniques on prosthetics does not give the same accuracy. The main reason behind preparing this review paper is to show existing development achieved over the years on real-time usability of hand prosthesis. More focus is given on PR classification techniques, feature extraction technique, embedded processor, virtual reality and other factors (sampling frequency, window length). Many review paper has been published on EMG-PR, but in our understanding, this is one of a few review papers on real-time EMG-PR control of hand prosthesis. 

**Figure 1:**
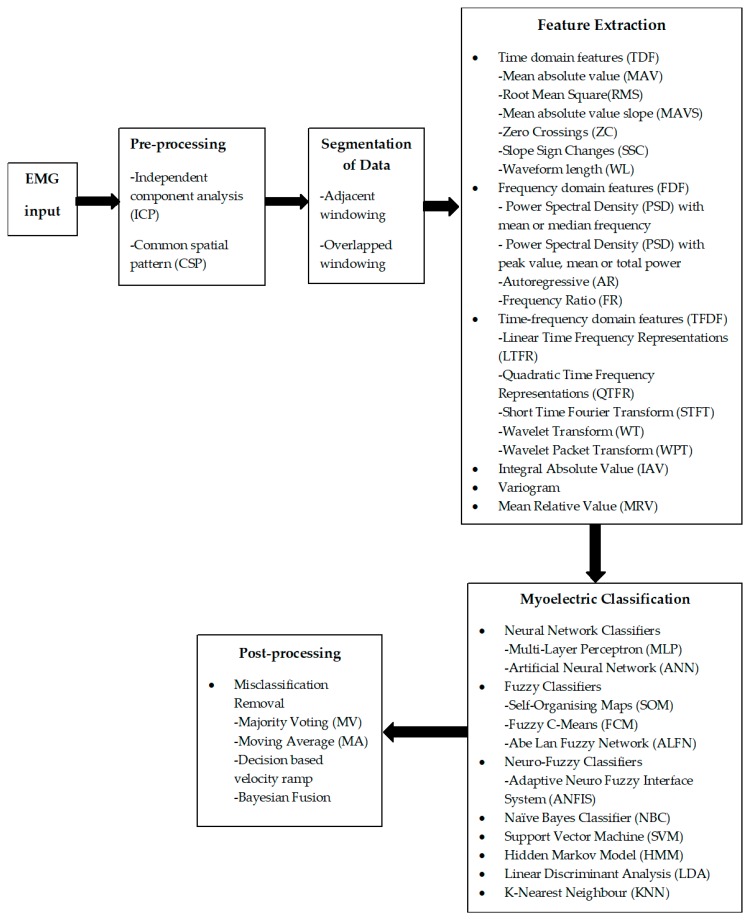
General pattern recognition schemes [11,26,27]. EMG: electromyography.

Firstly, the key ideas related to the EMG characteristics are discussed in brief on the introduction. The process of pattern recognition techniques is presented in Figure 1 and then discussed. Following this, we will explore the key techniques and research ideas related to the real-time myoelectric pattern recognition and control. These key ideas were established through a literature survey that attempted to capture the major achievement in the field of classification to obtain real-time control of prosthesis, real-time with embedded PR based prosthetics and real-time using virtual reality. In the final sections of the paper, we speculate on future research directions in this field based on our critical analysis of the current state of the art.

## 3. Pattern Recognition-Based Myoelectric Control

Myoelectric control systems can be classified as a pattern recognition control system and a non-pattern recognition control system. The non-pattern recognition control includes onset analysis, proportional level control and threshold level control. All of them are easy to implement in a real system but are limited in their degrees of freedom. From the early 1960s onwards, pattern recognition-classification techniques attracted the attention of the research community working on controlling artificial limbs. This approach consists of segmentation of data, feature extraction, and classification of a set of features or patterns for the various mode of myo-activations [23,24]. Figure 1 shows some of the existing pattern recognition techniques used for myoelectric controls. Feature extraction and windowing are two different parts of the [25] segmentation of data. Several studies use a pre-processing stage before the feature extraction to avoid the preliminary level of inherent disturbances and electromagnetic interferences. The output of pattern recognition is categorised into different classes or labels based on the input feature extracted. The classes define the control of the actuator with a specific command. In the next (sub) sections, we explain the detailed steps involved in real-time pattern classification of EMG based prosthetic hands. For simplicity, we summarised all the results (processing steps) as (a) real-time collected data from amputee (Table 1), (b) real-time using embedded packages (Table 2) and (c) real-time with virtual reality (Table 3), respectively.

### 3.1. Pre Processing of EMG Recorded Signal

Recorded EMG signal is characterised by many interferences, such as signal acquisition noise, electromagnetic disturbances, signal instability and motion artefact due to electrodes and cables. Pre-processing is the very first step of pattern recognition techniques regarding proper signal analysis and minimizing the inherent interferences [28]. It should be noted that Independent component analysis (ICA) and Common spatial pattern (CSP) are used as a Pre-processing (filtering) and dimensionality reduction (after feature extraction). 

### 3.2. Segmentation of Data

The results obtained from the Pre-processed EMG signal (random nature) is not regarded as a useful input in the pattern recognition technique. Thus, to extract the descriptive features, the window (segmentation) of the Pre-processed data is required. There are mainly two different types of windowing techniques proposed: overlapping window and non-overlapping (adjacent) window. In overlapped windowing technique, the former window overlaps over the current window with increment timeless than the window length itself [11]. The window length should be selected properly in real-time, which could deliver an acceptable delay. Larger window length would provide high classification accuracy but delay in the classifier’s decision. The different optimal length used in various works of literature has been reported in Table 1, Table 2 and Table 3. 

Segmentation of EMG data (using windowing) helps in estimating the intended motion for the myoelectric classifier. It helps in the decision making of intended motion while new data are being acquired. Englehart and Hudgins [29] used an adjacent, disjoint analysis window length equivalent to 0.25 × Sample frequency (SF) (256 ms for SF of 1000 Hz) for continuous myoelectric classification [30]. They also demonstrated that the data segment length of 0.125 × SF (128 ms for SF of 1000 Hz) or even less as 0.03125 × SF (32 ms) could be considered, without much reduction in accuracy for the continuous segmentation of steady-state signal. As with the advanced real-time computation facility and high-speed processors, data processing could take less than 5 ms, thereby classifying data segment length could vary from 32 to 25 ms. In this approach, with the time increment less than segment length, the new segment could slide over the current segment. The segment length must be higher than the processing period because the mainframe feature set had been calculated and must take a choice earlier to the next segment. Thus, normally, the denser yet semi-class decisions are made through small segment increments that help to improve response time and accuracy [31].

### 3.3. Feature Extraction

Generally, EMG features are extracted in the form of time-domain (TD), frequency domain (FD) and time-frequency domain (TFD). In the TD, the features are extracted from the variations of signal amplitude with time [32] as per the muscular conditions. Unlike time features, the frequency domain uses the power spectrum density of the myosignals for extraction [33]. On the other hand, the combined features of time and frequency domain are used for time-frequency extraction (examples such as short Fourier transform and wavelets). The studies based on feature extractions proposed across TD, FD and TFD shows the best results using the TD EMG feature. Hudgins [34] proposed the four different time-domain features (MAV, WL, ZC, SSC) [35] for feature extraction from EMG, and it is the most adopted one to date in the field of myoelectric pattern recognition [11]. Willison amplitude (WAMP) [36], Autoregressive (AR) model parameters [37] and time domain-auto regression (TD-AR) are also used to extract feature information. In comparison to other feature extraction methods, such as Fourier transform and Wavelet Transforms (WT), TD-AR features have achieved higher classification performance for the detection of hand movements with EMG signals [37]. Lui and Huang [38] implemented a fourth-order AR model for the EMG feature extraction and showed better classification performance. This approach only includes the trained data (EMG pattern classes) and rejects all untrained data of the classifier. Some of the recently developed features are Wavelet packet transform (WPT) based features [11], short-time Fourier transform (STFT) [39] and EMG synergies by matrix factorization analysis [40]. STFT comparing to TD and fractal domain features state EMG signals better relationships with different muscles. 

Recently, time-dependent power spectral descriptors (TD-PSD) [11] were proposed, which consists of feature sets (wavelength ration, sparseness, irregularity factor and spectral moments (first, second and fourth)). TD-PSD with force level training shows more robustness of pattern recognition against force variation than most of the other feature extraction methods [41], such as reduced spectral moments by Vuskovic and Du (VD-MOM), AR+RMS, TD, wavelet and discrete Fourier transform (DFT) [42]. Khushaba [43] proposed a temporal-spatial descriptor (TSDs). EMG features set collected from several intact-limbed and amputees are accepted on multiple sparse and high-density (HD) for executing multiple degrees of freedom (hand and finger movements). Time-derivative moments (TDM) based feature extraction [44] is a novel feature set extraction proposed to enhance the performance of EMG-PR in upper limb motion classification. Furthermore, most of the previous studies had focused on time-domain features to reduce computational difficulty. In addition, it does not require additional levels of data transformation [45].

Usually, after feature extraction, dimensionality reduction (DR) is applied. DR is the process of removing the number of arbitrary variables under consideration by locating a group of key variables. When the information is liberally dispersed (scattered) due to the EMG classification, there may be a problem caused by the large variance of the EMG signal. Thus, dimensionality reduction methods can unite this information more effectively and solve the problem of feature dimension. Dimensionality reduction thus helps in saving the computational cost and reduces the level of system complexity [46]. Uncorrelated linear discriminant analysis (ULDA), principle component analysis (PCA), and orthogonal fuzzy neighbourhood discriminative approach (OFNDA) are common dimensionality reduction (DR) techniques used to reduce the feature space.

### 3.4. Myoelectric Classification

The next stage, followed by feature extraction, is feature classification. The information gathered during feature extraction is fed into the classification stage. A classifier should be able to classify the pattern efficiently in less time to meet the real-time constraints of the prosthesis. Notably, only a few numbers of studies have compared the potentiality of classifiers to meet real-time control. 

The myosignals pattern classification for explicit movements is more focused on the extraction of activities from arm muscles. For amputees, due to their amputations, only a few muscles will be present in the residual limb to extract the feature of signals. For instance, in the case of transhumeral amputation, the availability of forearm muscles is completely unavailable. Most of the pattern recognition studies are, therefore, concentrated on trans-radial control. However, several studies tried to classify finger movements using multiple features and classifiers, such as multi-layer perceptron and neural network. Usually, in EMG-PR, the classifier performs well on trained (actions) classification patterns. To improve the robustness of PR systems, the untrained classification pattern is also equally important. Furthermore, from the past few years, the detection of untrained actions (novelty detection) has also been studied. In order to solve the problem of novelty detection [47], different methods were proposed and studied, such as the ensemble of one-class support vector data description (SVDD) [38], and modified boosted random forests [47].

Although various classification methods are available, classification algorithms include two main trends: Statistical (LDA and SVM) and Neural (MLP and ANN) [48]. The conventional PR method also depended on KNN and LDA algorithms to categorise arm motions into different pattern classes. LDA scheme is one of the most adopted classifiers in the implementation of myoelectric control. LDA classifier has shown high classification accuracy, and it is very simple to implement [49]. In KNN feasibility and precision check of classifying features have done for diverse time windows such as EMG histogram and noise levels [50,51]. In 2001, instead of concentrating fully on the classifier like KNN or LDA, some authors demonstrated that the pattern classification and its accuracy are more deeply depend on the selection of features [39,52]. As the input data are suddenly changing myosignals, they have the downside of immediately switching the control from rest to contraction. This prohibits changeover of the feature set from one to another in less time and an efficient manner.

Moreover, it delays the coordination between multiple tasks utilizing large degrees of freedom in real-time. As a solution, the wavelet packet-based feature set can classify myoelectric activity in real-time where the data streams are continuously exhibiting superior performance. A research work then started on the KNN classifier with a genetic algorithm, upon the trans-radial muscles having the potential to control a multi-fingered prosthetic hand. 

Another classification technique mostly used for the EMG signal classification is an artificial neural network (ANN). An ANN is easily trainable and has the capability of modelling both linear and non-linear data [49]. The SVM classifier, due to its kernel-based characteristics, has gained wide application in the field of myoelectric control. SVM is most popular for performing classification as well as regression using machine learning tasks. SVM is an advanced statistical learning approach providing an accurate and optimal solution in a short time. The different classifiers used in various works of literature have been reported in Table 1, Table 2 and Table 3. 

Although a lot of developments and progress were made in the field of EMG-PR, the development of a DOF prosthesis that could aid in simultaneous prosthetic control is still a challenge. Based on the literature studies, it indicates that classification accuracy can be increased with appropriate use of EMG channel and feature set.

### 3.5. Post-Processing for Upper Limb EMG 

To overcome the limitations of conventional EMG control [11] post-processing has been proposed. Furthermore, the post-processing stage is next to the classification level for the removal of errors and misclassifications. The control performance of a multifunctional prosthesis in a practical and laboratory setting will always show various disparities. To minimise this classification error due to unintended actions during the real-time applications, Simon et al. [53] introduced the practice of decision-based velocity ramp functions as a post-processing method. This function attenuates the speed of action soon after the classifier decision is altered. Moreover, post-processing approaches give a smooth state transition from the current motion class to the changeover state. The greatest advantage of this is that it could be combined with the multi-level real-time continuous control. Some of the other post-processing techniques are Moving Velocity [30], and the majority vote [39,54,55]. The majority vote [56] has also shown improvement in real-time EMG-PR control of hand prosthesis.

All advances in pattern recognition schemes with multiple input channels have improved the overall classification accuracy for multifunctional control. Pattern recognition methods inherently use sequential control, which requires sufficient windows (intervals) to extract useful classification features without delay of response time. When the processing window decreases, the performance window decreases significantly. Generally, with a normal pattern recognition algorithm, simultaneous and proportional control is difficult to achieve. For instance, to grab an object—closing of fingers together with pronation—an additional combination of classification features is required. This may increase the number of patterns needed to be trained, which in turn creates an undesirable increase in response time.

Moreover, pattern recognition does not provide proportional control, which is critical for optimizing the response time. As a classifier is a binary decision control, it cannot influence the speed or strength of prosthesis that requires an additional proportional component to the control of signal. This will make the system more complex and decrease overall power [57].

To overcome the need for a complex device and to provide proportional and simultaneous control, regression is one of the commonly used methods [47]. The regression approach can evaluate number of control signals continuously from the EMG signal directly. For example, if one control signal assists wrist rotation then the other control signal evaluates hand opening. Moreover, this approach provides more user-friendly and spontaneous control of the prosthesis. Some of the regression approach used for the control (movement) of prosthesis are linear and non-linear regression [16], ANN [58] and non-negative matrix factorization [40]. The regression method has thus far presented promising results, permitting direct and spontaneous control to the user and further developments are likely to reinforce the robustness of regression approach. 

## 4. Real-Time Application of Myoelectric Prosthesis

The high functionality (multiple DOF) and high accuracy are achieved on testing offline and real-time collected data from amputees. However, when tested by amputees or patients for real-time usability, it does not give the same level of accuracy. Moving from the virtual environment to the real world requires the implementation of prosthetic devices. One of the major challenges that influenced the usability of the prosthesis is the lack of robust and a portable embedded system to implement the EMG- PR algorithms, other challenges include the design of dexterous prosthetic hands, multichannel electrodes placement, compensation between power consumption, small size, etc. [59]. Various hardware has been implemented to develop prosthetic hands for persons with disabilities. Hardware chips are designed for filtering EMG signals and other applications such as grasp detection and human-computer interventions to obtain an accurate signal for prosthetic arm control. Furthermore, virtual environments allow the user to practice different controlling gestures that the designated prosthetic device supposed to control in real-world [60]. Some of the experimental outcomes on the real-time collected data from amputee, real-time with embedded packages and real-time using virtual reality environment are discussing in the following Section 4.1, Section 4.2 and Section 4.3, respectively. 

### 4.1. Real-Time Collected Data from Amputee

**Figure 2:**
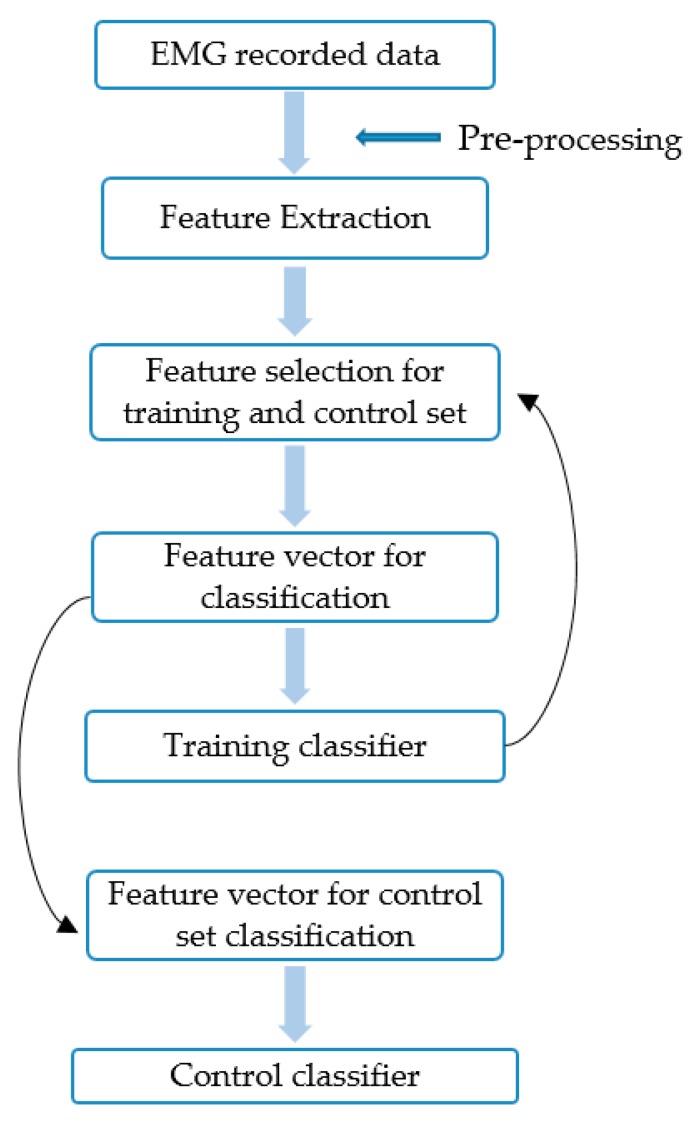
General pattern followed by the EMG-PR for real-time collected data from amputee.

Several research studies on able-bodied and hand amputees have been done to evaluate the consequences of arm position variation on EMG-PR classification performance. Offline classification accuracy/errors have shown that arm variation affects the classification performance. To reduce such effect of arm variation, various classification techniques [61] have been proposed. Similarly, classification accuracy identifies the desired movements from several classes of motion. Some of the previous research studies had shown that offline classification has not a good correlation with real-time performance of EMG-PR [61] control of the prosthesis. However, some of the recent classification accuracies on the real-time performance of amputee data are explained in this section. Nearly all EMG-PR control for real-time collected data from hand amputee followed the same stages to operate. The features are extracted from pre-processed EMG data. From the extracted data, the feature is usually selected for training and control set. Then, the classification technique is applied for training classifiers and control set classifiers. The general algorithm of this whole system is shown in Figure 2. 

In 2003, Karlik et al. [62] conducted a study on the classification of myoelectric signals for precise overall control of multifunction prosthesis using Fuzzy clustering neural network architecture. The author also compared the accuracy of multi-layer perceptron (MLP) having a back-propagation algorithm and the new fuzzy clustering neural networks (FCCN). The fuzzy clustering involved the division of input data into several fuzzy parts that intersect each other and thus defined by membership grade [0,1]. An algorithm was proposed to implement these fuzzy clustering that minimised the cost function. A comparative assessment shows that using FCNN provides more reliable results than MLP. The FCNN achieved 98% accuracy with half training time than that of MLP. Later, in 2005, a promising method by Chan and Englehart [63] added into the row of the continuous controllers. The new method followed a hidden Markov model (HMM) as the data segment classifier. The HMM classifier was a suitable probabilistic approach for pattern recognition at that time due to the resilience to sequential myosignal variations. For a four-channel six function design, HMM has more performance accuracy (94.63%) than MLP with a good level of robustness and quick response. 

Al-Timemy et al. [37] proposed a process for the classification of finger motions for dexterous control of the myoelectric prosthesis. The myosignal was recorded from six traumatic below-elbow amputees. TD-AR features were used to extract useful information from the segmented EMG window. To find the best match of features reduction (to reduce computational power) and classifiers, two different features reduction tools (PCA and orthogonal fuzzy neighbourhood discriminative approach (OFNDA)) and classifiers (LDA and SVM) were combined to make four different forms. The results show the high accuracy with OFNDA and LDA. 

Furthermore, the studies show that feature reduction plays an important role than a classifier to achieve high accuracy with multi EMG channels. In 2013, Pan et al. proposed a solution for partial hand amputees with a functional wrist to predict the finger joint angle using EMG [64]. The experiment was performed on two amputees. EMG signal was recorded from eight targeted muscles and was sampled at 2000 Hz frequency. TD feature sets were fed to the LDA classifier to identify seven different static wrist positions. A switching rule, including LDA classifier and 14 state-space models, was proposed for continuous decoding of finger joint angles. The average classification error rate (CER) was 6.18%, which demonstrates that forearm movements and the continuous movement of the finger can be easily classified. Similarly, in 2016, Ganesh et al. proposed the combination of ICA and Icasso to minimise the number of EMG sensors and increased robustness of myoelectric control [12]. 

Early in 1993, [34] an experiment was performed on one amputee using the Hudgins feature set. These features classified using ANN classifier with one EMG channel proved that the EMG signal shows deterministic structure during the beginning of muscle contraction. Riillo et al. [65] proposed an optimization methodology of sEMG based hand gesture classification using pre-processing techniques such as PCA (unsupervised) and CSP (supervised). One trans-radial amputee (right-hand below-elbow amputee) participated in the experiment. TD features were extracted from segmented data (using overlapped windows). Similarly, three classifiers (LDA, SVM and ANN) [65] were tested by assessing the average accuracies of each time window. The study shows that the best results obtained for the real-time system were using the ANN classifier. 

Another research work extended the classification control using a support vector machine (SVM), obtained high accuracy (92–98%) with less training time [66]. Stango et al. [67] used the SVM classification technique, followed by variogram features. The variogram is a measurable degree of spatial correlation. The experiment was performed on one trans-radial traumatic amputees. The main purpose of this experiment was to analyse the spatial features of HD EMG-PR for myoelectric control. Variogram features maintain a good classification accuracy without retraining even if the EMG channel is eliminated during the experiment phase. Hence, the study shows spatial proposed improved the robustness of EMG-PR. In [68], the effectiveness of using twin SVM (TSVM) in multi-class prosthetic control with unbalanced datasets was demonstrated with the RMS value (feature). 

The summary of the comparison of some of the EMG-PR classifiers using real-time amputee data is shown in Table 1. All the achieved accuracy was demonstrated only in ideal research settings.

Among the many classifiers (Figure 1) in myoelectric control, LDA seems to be widely used classifiers. On the other hand, SVM and KNN, due to their kernel trick characteristics and non-parametric nature [51], respectively, have equally been used widely. Though the better performance was achieved with many classifiers, high-density surface EMG is impractical to use as a source for real-time control. 

### 4.2. Real-Time EMG-PR with Embedded System

**Figure 3:**
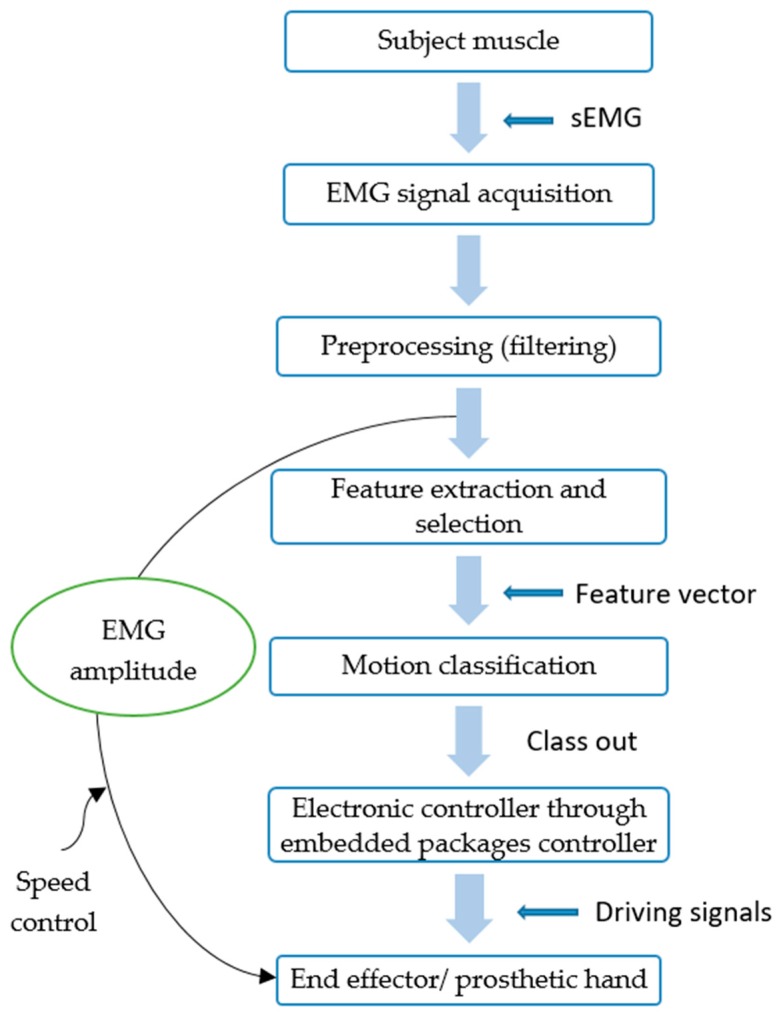
Real-time EMG-PR with embedded system.

An embedded system was customised to perform a specific task and function often with real-time control. This system was mainly based on microcontrollers or microprocessors. Real-time embedded for the EMG-PR for hand prosthesis can be enhanced to reduce cost, size and increase the reliability and performance of the prosthetic device. Almost all of the real-time EMG-PR control using embedded systems followed the same stages to operate, as shown in Figure 3. Firstly, the sEMG signal is recorded from the subject muscle using electrodes. Then sEMG signal acquisition takes place. This signal goes through pre-processing techniques. The features are extracted and selected from the pre-processed signal. Once the selected features are classified, the command was sent to the embedded controller to control the end effector. 

Wirta et al. [73] first reported the used of embedded myoelectric pattern recognition system in 1963. The robotic arm was developed, and Discriminant analysis was chosen as a classification technique. After a few years during 1996, a real-time EMG-PR was proposed with a digital signal processing (DSP) (TMS320C31) based system having a modified maximum likelihood distance (MMLD) classifier. Four able-bodied and two quadriplegic subjects volunteered and were designated with five motions of neck and shoulders. The total response time for EMG discrimination was 0.17 s, and it achieved a 95% mean discrimination rate [74]. An analogue integrated circuit for the wireless transmission of physiological signals designed by Yeng et al. [75] focused more on the transmission system, not on the implementation of the prosthesis. In 1999, an Evolvable Hardware (EHW) chip for myoelectric artificial hands was developed to serve as a standard tool for hardware validation [76].

To access the computer for limbs disabled through their remaining muscles, a real-time assistive device was designed in 2007 with PR of EMG signals [77]. The signals were measured from the muscles of the lower arm of the subject during different wrist motions. The obtained signals were filtered, and a supervised multi-layer neural network trained by a backpropagation algorithm was used for the classification of the user’s movement and clicking of a cursor. The drawback of that article was that the researcher focussed more on the qualitative evaluation of performance than presenting the control implementation. Similarly, Anbin et al. [78] proposed the novel combination of signals (EMG and inertial measurement unit (IMU)) to be used for mouse controller (cursor movements). LDA classifies the EMG data into several groups of 128 ms time window and 32 ms increment window, which correspond to the pre-defined computer mouse operations. The results showed an accuracy of 88%. 

In 2007, Bitar et al. [48] explained in detail the design of portable Musical Instrument Digital Interface (MIDI) using a continuous wavelet transform (CWT) decomposition and SVM. A low-complexity portable dsPIC33FJ256GP710 embedded system was designed that collects and classifies EMG signals. This embedded system is quite inexpensive and consumes less power. The output from four-channel was sampled at 1 kHz frequency using the dsPIC’s on-chip A/D converters. The channel window (fixed-length windows of 0.6 s with 0.3 s overlap) was normalised by its respective power. The CWT coefficients were computed for each and every channel separately, and the desired features were extracted. Finally, six class classifications were performed using the SVM classifier, and the decision of the classifier transmits the result as labels in real-time using Bluetooth to a remote interface. Moreover, to control a MIDI-enabled device (mechanical prosthetic hand), these labels were then converted to MIDI commands. The experiment showed an achieved 91% accuracy.

Ke et al [59] presented the latest progress on EMG-PR control of a prosthetic hand. EMG signal was acquired using an armband with eight-channel electrodes. A powerful embedded system was introduced to deal with the decoding algorithm of EMG signals. These real-time surface myoelectric signals decoding and EMG training (onboard) were incorporated in the embedded system to control a prosthetic hand of six DOFs. The result showed that the possibility of speeding up the movement of a PR prosthetic arm, making it more suited for daily application, is promising.

In 2013, Xiaorong et al. [79] proposed a first real-time EMG-PR self-recovery classification using a cumulative sum algorithm (CUSUM) detector. Forty-eight motion artefacts were introduced on 12 real-time testing trials. CUSUM detector successfully detected the 43 artifacts, which lead to 93.5% of the elimination of misclassification caused by motion artefact. Similarly, in 2015, Ann et al. [80] compared the non-adaptive (conventional) and adaptive control (real-time) prediction learning. The experiment was performed on one trans-humeral prosthesis and three able-bodied subjects. EMG signal acquired using eight channels sampled at 1 kHz to classify the eight classes of motion. Subjects were asked to wear a Bento arm (anthropometric robotic arm), which consists of MTT (AX-18 smart robotic arm) incorporating five DOF. The result shows the adaptive control decreases the total switching time and improve myoelectric robotic arm during uninterrupted use by subjects (amputee and normally limbed). 

A few articles showed the real-time control of commercially available prostheses for finding the user experience with pattern recognition control. Understanding the patient’s experience can help clinicians and patients who choose prosthetic options. The commercially available EMG-PR control was interfaced with multiple degrees of freedom DEKA arm [81]. This study provided an extensive description of the user experience of operating a DEKA arm using EMG-PR control. The majority of the participants preferred the future prospective of EMG-PR as a control measure. 

Mastinu et al. [82] presented the real-time implementations of PR techniques on dysmelia subjects (congenital disorder). The subject was asked to use iLimb-ultra (Touch Bionics, UK) for five consecutive days during the experiment. This system is known as the artificial limb controller which includes a pattern recognition system. The classification accuracy and motion test of the system were compared with different classes on motion (open hand, closed hand, side grip, fine grip and pointer) individually. The real-time pattern recognition accuracy for motion test (subjects were asked to perform as directed on-screen) was higher than the classification or execution accuracy. 

Hargrove et al. [83] demonstrated the outcomes obtained from the commercially available prosthetic used by subjects undergone targeted muscle reinnervation (TMR). Subjects wore a commercially available prosthesis to perform different household tasks. A comparison of direct method and pattern recognition methods in TMR subjects were performed, and statistical significance of both methods was evaluated. Users performed well with pattern recognition incorporated devices. Additionally, among the eight subjects participated, seven preferred pattern recognition control. Some of the papers related to the real-time with embedded packages are summarised and shown in Table 2.

Among many classifiers (NN and SVM), LDA is one of the most used classifiers for real-time embedded systems. LDA’s main advantages are its simplicity of implementation in an embedded processor. Although many studies have been done and the embedded system has been implemented to develop the prosthetic of a lost limb using EMG-PR control, a major issue of achieving natural and reliable control of limb remains unsolved.

### 4.3. Real-Time Using Virtual Reality 

**Figure 4:**
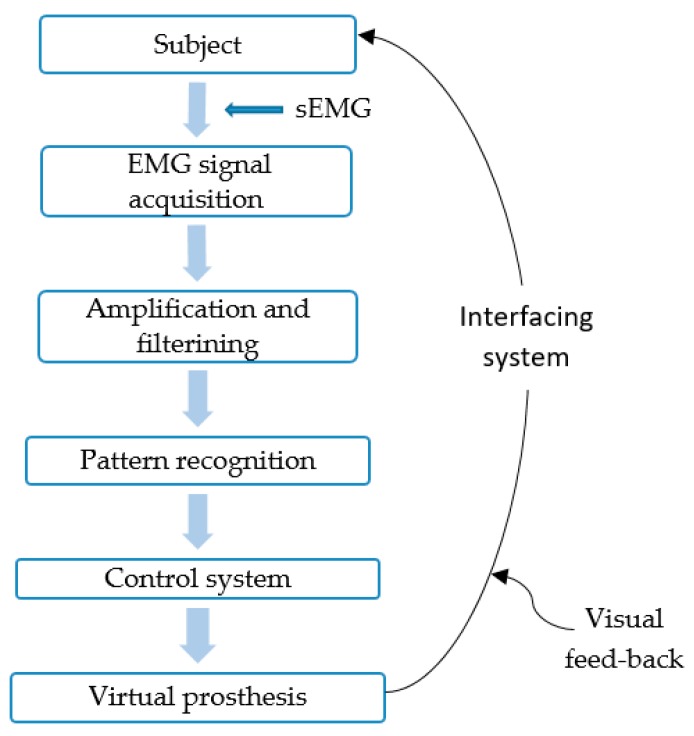
General representation of virtual prosthesis process.

To be able to control virtual prosthesis and to become familiar with a real-time prosthesis, voluntary muscle contraction control is very important; this can be done by using a visual feedback system. The improvement of learning depends on the user and visual feedback system, thus, the feedback system must allow the user to learn new tasks using their muscles [87]. Most of the virtual prosthesis followed the same stages to operate. Firstly, the EMG signal acquisition takes place using electrodes on the residual muscle of amputees. Then, the signal is amplified and filtered to acquire the myoelectric signal to be used. The interface between the virtual system and acquisition of myoelectric signal is created, which consists of isolation, pre-processing of the signal in the hardware, personal computer (PC) communication, communication between PC and MATLAB, processing in software and communication between MATLAB and virtual world [87] (part of MATLAB). The general idea followed by most of the virtual prosthesis is shown in Figure 4. 

The continuing the examination of real-time control of prostheses using the myoelectric signal resulted in a robust scheme pattern recognition [29]. Twelve subject data from four channels were used for real-time control. Unlike the traditional methods involving transient control, which requires initiation from rest, a continuous stream of class decisions was delivered to the prosthetic device. Pattern recognition was performed on sliding time windows with 256 ms in duration and with the LDA classifier. The continuous decision (intended motion) permitted intricate classifications involving multiple joints without disruption. The continuous classifier performs very well with a significant gain in accuracy and response time over a wide range of analysis window lengths if accompanied by majority vote post-processing. Moreover, the control scheme required minimal storage capacity.

Ann et al. [88] presented that the target achievement control (TAC) test in the virtual environment provides a good platform for PR control practice and testing. In a TAC test, virtual prosthesis moved from an inactive position to the target position. In 2015, Martina and Haripriya [89] constructed a prototype using sEMG signal to record the data from the brachioradialis muscle of forearm to control the movement of powerpoint slides transmitted in real-time. Furthermore, Agamemnon et al. [90] performed an experiment on 20 able-bodied and two amputees to find the outcome of two sensors (sEMG and inertial measurement (IM)). Twelve electrodes were used to acquire sEMG signal in a sampling frequency of 2 kHz. Feature sets such as MAV, WL, 4AR and logVar were extracted using a sliding window of 256 ms with 50 ms increments. Two sets of the experiment (offline and real-time) were performed. Real-time prosthetic hand control was based on offline observation. Touch Bionics ’robotic hand’ have been used for real-time performance. It shows that the combination of both IM and sEMG improved the classification performance of a prosthetic hand. Additionally, the use of IM and sEMG reduce number of sensor require to achieve high level of accuracy. Yanjuan et al [61] investigated that both offline motion classification accuracy and real-time motion completion rate are important to assess the performance of EMG-PR control. 

Identifying multiple DOF (hand movements) using a few EMG sensors is one of the necessities for developing high levels of usability prosthetic hands. Trongmun et al. [91] present a signal processing technique that classifies 17 spontaneous classes of motion from EMG signals using spectral features and an ANN. Online classification experiments were performed on twelve subjects (seven male and five female) to assess the reliability of the proposed method. An overall correct classification rate of 83% was achieved, showing the ability to classify 17 movements from six EMG sensors. Moreover, the classification of nine movements could achieve accuracy of up to 92%. EMG pattern classification has been widely studied to decode user-determined for intuitive prosthesis control. 

The significant breakthrough was occurred with the introduction of surgical procedure to improve the control of hand prosthesis known as targeted muscle reinnervation (TMR) [92]. The real-time and offline performance of EMG-PR with TMR patients was presented using a generic electrode grid. Four amputee subjects (two trans-humeral, two shoulder articulation) that underwent TMR surgery participated in this study. In a real-time virtual analysis as well as offline classification, a generic grid-like electrode performed better than the control site (specific site for electrode placement). Although TMR has the potential to provide advanced control of wrist and grasp patterns for myoelectric control, the concept has not yet been a success in implementing it to multiple DOFs for the prosthesis. 

For assessing the real-time PR control of TMR based multifunction prostheses, Todd et al. [86] showed the performance outcome based on motion (selection time, completion time and completion rate). The experiment was performed on both virtual and real prosthesis. The performance was first ascertained by training and testing with a virtual multifunction prosthesis. Later on, the experiment was carried on three TMR patients with upper-limb prostheses. The mean classification accuracy of 88% with standard deviation (SD) of 7% for patients who had undergone TMR surgery and 97% with standard deviation (SD 2%) for control participants was achieved. Furthermore, the summary of some studies based on real-time using the virtual reality environment is presented in Table 3.

## 5. Discussion

The first pattern recognition control scheme was developed in the late 1960s. By the 1980s, the approach was more refined by extracting features using autoregression from a smaller number of input channels. This allowed greater accuracy (nearly 86%), but was unable to achieve that in real-time. At the beginning of the 1990s, pattern recognition and its accuracy were improved further with artificial neural networks. Then, the methodology was shifted to the analysis of real-time scenarios with a continuous shrinkage to permit precision of roughly above 92%. The inclusion of real-life constraints and reduction of dynamic error were large discussions after the late 1990s. Since then, most of the studies attempt to achieve a perfect natural level control in myo prosthesis by the selection of appropriate classifiers and post-processing techniques. It is obvious that the popularity of pattern recognition methods keeps on increasing, and the research studies are evolving into a more natural control of artificial arms. A sudden increase in pattern recognition control can be visible from the year 2000 onwards. Though there are some fluctuations in the level, with the change in computing capability of processors, the interest in research on pattern recognition has risen and shown major turn since 2010.

There are still many challenges to implementing real-time prosthesis, mainly in a wearable embedded system. First, a solution scheme involves a re-training PR classifier. Presently, this process includes the restructuring of the training feature matrix, the estimation of variables in the pattern classifiers, and then forming new organization of the testing feature vectors. It is unknown if the embedded system can control all this approach fast enough for each decision. Second, many components are incorporated into the EMG-PR algorithm; the interaction between the components and the precise time control is critical.

At last, it requires a compact combination of all components of the embedded PC. The system requires to provide the interfaces needed for the collection of data, sufficient computing power for decision making in real-time, effective memory management and low energy utilization [79]. All of the above-mentioned challenges are less explored.

Most of the above said articles tried to analyse and use repeated data by setting ideal clinical conditions for classification error and accuracy. Moreover, the real-time articles mostly tested their results with able-bodied subjects. In real amputee life, some unwanted, unrealistic repeatable contractions can be observed from myo-signals during classifier learning. Those considerations were the least discussed and identified. When a user is asked to perform several activities under real-life conditions, such as varying size loads, orientation and weather, the classification error in the real-life scenario is high from their equivalent able-bodied subject. It is also clear that pattern recognition systems have yet to obtain an extensive application for numerous reasons such as (1) the absence of good user interface, (2) uncertainty in classification accurateness and control and (3) variations in patterns over the period. The previous studies have shown practical achievements for the control of ULPs. Together with this achievement, the advancement of inaccurate classification and speed from a reliable command method is enough to process with less time, less error and minimum mental effort. While many classification schemes have been analysed, generally, the feature identification methods are stuck to time-domain features and are paired with LDA classifier. 

Moreover, none of the pattern recognition systems have been found to be 100% precise. The wrong classifications need to be alleviated to make a myoelectric pattern recognition control as a valid choice for an amputee. Otherwise, users become frustrated, as they are unsuccessful at completing a task due to unintended prosthesis movement. Ultimately, this can lead to the rejection of the device itself.

## 6. Challenges and Future Prospects

With technological advancement, purely aesthetic orthopaedic prostheses have gained more and more functionality over the years. Although the prosthesis nowadays provides a lot of movements (DOF) for amputees, there are still some challenges that need to be fixed for the real-time EMG-PR control for hand prosthesis. 

The real-time usability of available multiple DOF prosthesis is impacted by various factors such as intuitiveness of device, comfort, appearance, function, durability and cost. Furthermore, there are some other compounding factors, which are explained in Table 4.

### Future Prospects-Implementation of Real-Time EMG-PR Control

- Electroencephalogram (EEG), and ECog (Electrocortocogram) measures brain signals, and they could be used to supersede EMG for prostheses control. ECog electrodes are invasive as they are placed directly inside the head, whereas EEG electrodes are non-invasive, as they are positioned on the scalp area [100], where information regarding the targeted body movements are measurable [101]. EEG and ECog have currently found an application as a brain-machine interface [102], and in theory, can control the movement of the prosthesis similar to the EMG. In other words, while EMG measures the electric current from muscles and provides the control signal according to the action intended by subject [103], brain-machine interface decodes the electrical signal generated from the brain and converts them to the control signal for the control of prostheses [104] without using a muscle as an intermediate [100]. Unfortunately, due to the invasiveness (ECog) and the problem associated with electrodes montage stability (EEG), generalised poor signal-to-noise ratio (SNR) and the poor spatial resolution of the signals, not to mention the discomfort related to the need of having multiple devices over the subject’s body (i.e., head and limbs), we believe that, at present, these devices may be better suited for patients with spinal cord injury where voluntary EMG signals may be not available. It is necessary to mention that another issue often related to the use of brain signals to drive external devices is the need of extensive training [105,106] and poor performances of the brain to computer interface.
- The prosthetic control unit should be increased, and appropriate pattern-recognition should be used for proper handling of the prosthetic device.
- The prosthetic device should be developed using low-cost materials, affordable to all amputees.
- Intuitiveness can be developed by extracting the signals using ultrasound imaging [107], force myography (FMG), TMR and Implantable myoelectric sensor [108].
- One possible way to minimise or eliminate this drawback of EMG interferences is to develop an electromagnetic shielding technique [11] and implement the best filtering strategy.
- Rather than depending on existing proposed training, an intelligent adaptive prosthetic system should be developed and implemented. An intelligent EMG-PR system requires to represent a data stream accurately in real-time. It shows a possible way to restrict the deficiency in the prosthesis market. With such developments, users’ expectations can be met, and thus, device adoption for everyday use can be increased.
- Feature extraction is a core of conventional EMG-based pattern recognition control. To achieve the real-time usability of prosthetic, issues related to the feature extraction should be addressed. Deep learning (machine learning method based on ANN) may be one possible way to solve the problem of feature extraction [11]. Thus, more research on deep learning in pattern recognition-based prosthesis control should be conducted.

## 7. Conclusions

This review paper presents a brief introduction to EMG-PR techniques and explores the work done on real-time (amputee data, embedded and virtual environment) myo-activated prosthesis based on pattern recognition control over the years. Through the literature survey, some of the key techniques required for the improvement of existing real-time application of EMG-PR for hand prosthesis are presented. Although the perspective of intelligent pattern recognition control methods for the multiple degrees of freedom for hand prosthesis has been well investigated, their real-time usability is still being challenged by a number of compounding factors. Natural neuromuscular control of prosthesis should be proportional and investigate multiple degrees of freedom. However, while reviewing the existing literature, we have observed that the majority of real-time prosthesis uses EMG, i.e., multiple channels targeting multiple residual muscles to generate multiple synchronous control signals. The challenges are even greater than a single degree of freedom due to the proximity of the muscles/electrodes, etc. This should be well investigated in the future for real-time scenarios. Furthermore, to achieve real-time usability, appropriate design of the prosthetic device, virtual training, feature extraction and classification techniques should be properly investigated and implemented. 

## Figures and Tables

**Table 1:** Summary of various individual classifiers and combined classifiers tested on amputee data ^1^.

Pre-Processing	Segmentation/Window Length	Feature Extraction/DR	Classification	Post-Processing	Classes/EMG Channel	Accuracy
N/A	256 ms overlapping 32 ms	TD, 6AR, RMS/PCA, ULDA	KNN, LDA [69]	Majority vote	7/57	>97%
N/A	200 ms length with 50 ms increment	6AR, RMS, IAV, ZC, WL, SSC/OFNDA	LDA [37]	N/A	12/11	90%
N/A	200 ms with 5 ms increment window	MAV, ZC, WL, SSC/N/A	LDA [64]	N/A	7/7	95.64%
ICA	250 ms overlapping window with 64 ms increment	4AR, RMS, MAV, ZC, VAR, WL/ULDA	LDA [12]	N/A	12/11	>90%
CSP	300 ms with 75 ms of delay between the overlapped window	M, RMS, WA, SSC/PCA	ANN [65]	N/A	5/6	92.04%(PCA) 93.4%(CSP)
N/A	Window set to 4500 and window shift 50	Variogram/N/A	SVM [67]	N/A	7/48	81.6%
N/A	256 ms with window shift 32 ms	WL/N/A	NN [70]	N/A	4/6	An average RMS error=0.16 for 4 patterns
N/A	200 ms sliding window	RMS, log(rms)/N/A	Fuzzy c- means clustering [71]	N/A	4/3	87.5±13%
N/A	200 ms with an increment of 75 ms	RMS, WL, ZC, SSC/N/A	LDA [72]	N/A	6/8	>91%

^1^ Table omit the results from able-bodied subjects.

**Table 2:** Summary of the real-time controller in an embedded package.

Pre-Processing	Segmentation/Window Length	Feature Extraction	CLASSIFICATION	Post-Processing	Classes/EMG Channel	Sampling Frequency	Processor
N/A	N/A	N/A	MLNN [77]	N/A	6/4	1 kHz	PCI-6034e
N/A	600 samples (0.586 s)	CWT	SVM [48]	N/A	5/4	1024 Hz	dsPIC33FJ256GP710
N/A	Overlapped analysis window 160 ms with 20 ms increment	MAV, SSC, ZC, WL	LDA [79]	N/A	3/4	1000 Hz	M3-Microcontroller
N/A	300 ms with 200 ms overlap (100 ms increment)	MAV, SSC, ZC, WL	LDA [59]	N/A	6/8	200 K samples per sec	STM32F4072GT6
N/A	100 ms with 50 ms increment	MAV, SSC, ZC, WL	LDA [82]	N/A	5/7	1000 Hz	M4 microcontroller
N/A	250 ms	Integrate- EMG, RSS, INVAR	KFD (DR), RBFNN (classifi-er) [84]	Majority vote	9/8	200 Hz/channel	Arm Cortex—A53
N/A	200 ms with 175 overlap	MRV, WVL, ZC, SC, 6AR	LDA [85]	N/A	7/12	1000 Hz	Logic PD SOMDM 3730
N/A	150 ms analysis window with 50 ms overlap	MAV, ZC, SSC, WL	LDA [86]	N/A	11/12	1 kHz	USB-1616FS

**Table 3:** Summary of real-time analysis with a virtual prosthesis.

Pre-Processing	Segmentation/Window Length	Feature Extraction	Classification	Post-Processing	Classes/EMG Channel	Sampling Frequency
N/A	256 ms	ZC, MAV, SSC, WL	LDA [29]	Majority vote	4/4	1000 Hz
N/A	150 ms analysis window with 50 ms window increment	MAV, SSC, ZC, WL	LDA [88]	N/A	7/6	1 kHz
N/A	500 sample/s	N/A	NN [93]	N/A	8/17	N/A
N/A	32 sample hamming window with 75% overlap	PSDs	ANN [91]	N/A	17/6	200 Hz
N/A	Sequential analysis window 150 ms with a time increment of 100 ms (50 ms overlapping)	MAV, ZC, WL, SSC	SPC CC MPC [61]	Majority vote	7/16	1000 Hz
N/A	100 ms overlapping sliding window	MAV	Error-correcting output codes classifier [94]	N/A	13/15	2048 Hz
N/A	150 ms sliding window with 100 ms increment	MAV, ZC, WL, SSC	LDA [56]	Majority vote	7/6	1000 Hz
N/A	128 ms increment to 1024 ms	6AR and RMS	Linear regression cascade model [95]	N/A	3/6	1000 Hz
N/A	250 ms with 50 ms increment	6AR, MAV, ZC, SSC, WL	LDA [92]	N/A	(9-13-17-29)/(14-15)	1 kHz
N/A	200 ms sliding window	TD5 -MAV, SSC, WL, ZC	EASRC [28]	N/A	6/8	1000 Hz

**Table 4:** Some of the challenges of real-time EMG-PR control of hand prosthesis.

Challenges	Description
Comfort	The socket that is the part of the upper limb prosthesis may interfere with the elbow (a function of the residual joint). If the socket does not fit correctly, the patient may suffer from pain, sores and blisters. Such prostheses will be experienced as heavy and cumbersome [96]. Even some prostheses with appropriately designed sockets, face problems of heat, sweating and chafing.
Appearance	Most of the developed upper-limb prostheses do not look natural in appearance. Additionally, the user can find the prosthesis uncomfortable to wear. The user is still unable to control the multiple degrees of freedom simultaneously and consistently.
Function	Nowadays, upper limb prostheses perform almost all everyday activities. However, it remains challenging to obtain opening and closing positions of the hand from the residual limb. This is because residual muscles often used for hand prosthesis are biceps and triceps, which do not convey the information for closing and opening the hand [97].
Durability	Many of the upper limb prostheses are heavy and have short battery life.
Cost	Upper limb prosthesis costs around $50,000, which is quite difficult to afford by amputees from all over the world.
Technology	Developed prosthetic devices still lack intuitiveness and reliability between user motion volition and real motion of prosthesis. Similarly, much training is needed to operate those prosthetic hands.
Processing delay	The embedded processor used exhibits some delay (around 3 s), which halt the acquisition of EMG for that delay period.
EMG interferences	The transient changes in EMG often result from external interferences, changes in electrode impedance, muscle fatigue and electrode shift, among others. During practical use, this transient change arising from variations (long- and short-term) in the acquisition environment caused degradation of the clinical vitality of the device and limited its users’ adoption [11].
Electrode displacement (shift)	Electrode displacement occurs each time when users use a prosthesis, electrodes slightly reconcile in a different position relative to underlying musculature. When the user performs some task, due to the loading and positioning of limb, a movement of electrode occurs. Such an electrode shift can lead to a change in EMG characteristic (recording) of the limb, and thus, make it more difficult to decode the movements [98].
Amputee movement	EMG signal from the limb position is mostly recorded when the user is in a static position (sitting), but in a real-time scenario, prosthesis users have to use the device in different positions (walking, climbing stairs). However, the variation in limb position effect the classification performance of EMG-PR [99].
Muscle contraction forces	While performing everyday activities, the same limb assists different muscle contraction forces across different conditions. Thus, the variation in muscle contraction force occurs due to the same targeted limb results in myoelectric signal pattern classification inconsistency. Hence, it affects the EMG-PR control of prosthesis [24].
Limb position variation	Variation in limb position occurs while performing a different action in everyday life. For upper-limb amputation, the effects are seen on residual muscle (located in a prosthetic socket) from which the EMG signal is collected. Additionally, various limb positions lead to the variation in gravitational force, which leads to the displacement of target muscles. These factors cause variation in EMG signal pattern affecting the EMG-PR control of prosthesis performance.

## Abbreviations

ALFN	Abe lan fuzzy network
ANFIS	Adaptive neuro-fuzzy interface system
ANN	Artificial neural network
AR	Auto regressive
CER	Classification error rate
CSF	Common spatial pattern
CUSUM	Cummulative sum
CWT	Continuous wavelet transform
DFT	Discrete fourier transform
DOF	Degree of freedom
DSP	Digital signal processing
DR	Dimensionality reduction
EASRC	Extreme learning machine with adaptive sparse representation classification
ECog	Electrocorticogram
EEG	Electroencephalogram
EHW	Evolvable hardware
EMG	Electromyogram
FCM	Fuzzy C means
FCNN	Fuzzy clustering neural network
FD	Frequency domain
FDF	Frequency domain feature
FP	Feature projection
HD	High density
HMM	Hidden Markov model
IAV	Integral absolute value
ICA	Independent component analysis
IM	Inertial measurement
IMU	Inertial measurement unit
KFD	Kernel fisher discriminant
KNN	K-nearest neighbour
LDA	Linear discriminate analysis
LTFR	Linear time frequency representations
MA	Moving average
MAV	Mean absolute value
MDA	Multiple discriminant analysis
MIDI	Musical instrument digital interface
MLP	Multi-layer perceptron
MMLD	Maximum likelihood distance
MRV	Mean relative value
MV	Majority Voting
NBC	Naive Bayes classifier
NN	Neural network
OFNDA	Orthogonal fuzzy neighbourhood discriminative approach
PCA	Principal component analysis
PNS	Peripheral nervous system
PR	Pattern recognition
PSD	Power spectral density
QTFR	Quadratic time frequency representations
RBF	Radial Basis function
RF	Random forest
RMS	Root mean square
SD	Standard deviation
sEMG	Surface EMG
SF	Sample frequency
SNR	Signal to noise ratio
SOM	Self organizing maps
SPSE	Steady posture and steady EMG
SRC	Sparse representation classification
SSC	Slope sign change
STFT	Short time Fourier transform
SVDD	Support vector data description
SVM	Support vector machine
TAC	Target achievement control
TD	Time domain
TDF	Time domain features
TD-PSD	TD-Power spectral descriptors
TFDF	Time frequency domain features
TKE	Teager-Kaiser energy operator
TMR	Targeted muscle re-innervation
TSD	Temporal- spatial descriptor
TSVM	Twin SVM
ULAs	Upper limb amputees
ULDA	Uncorrelated linear discriminant analysis
ULP	Upper limb prosthesis
VD-MOM	Vuskovic and Du-MOM
WAMP	Willison amplitude
WL	Waveform length
WPT	Wavelet packet transforms
WT	Wavelet transform
ZC	Zero crossing
